# Fostering equity, diversity, and inclusion in large, first‐year classes: Using reflective practice questions to promote universal design for learning in ecology and evolution lessons

**DOI:** 10.1002/ece3.6960

**Published:** 2020-11-24

**Authors:** Laura Super, Analise Hofmann, Connie Leung, Mabel Ho, Emma Harrower, Najah Adreak, Zohreh Rezaie Manesh

**Affiliations:** ^1^ Forest and Conservation Sciences The University of British Columbia Vancouver BC Canada; ^2^ Cellular and Physiological Sciences The University of British Columbia Vancouver BC Canada; ^3^ Curriculum Developer Dalhousie University Halifax NS Canada; ^4^ Vancouver BC Canada; ^5^ Surgery Department Faculty of Medicine The University of British Columbia Vancouver BC Canada

**Keywords:** diversity and inclusion, equity, reflective practice, universal design for learning

## Abstract

Instructors can deliberately design for equity, diversity, and inclusion, including for large first‐year classes, and now instructors have added challenges given COVID‐19. Our paper explores the question: How do we integrate equity, diversity, and inclusion and universal design for learning (UDL) into first‐year, undergraduate ecology and evolution introductory lessons given the COVID‐19 pandemic? Given the large field exploring equity, diversity, and inclusion, we chose to focus on developing reflective practice question rubrics for before, during, and after lessons to encourage UDL for instructors, teaching assistants, and learners. We conducted a focus group within our team and discussed ideas related to online learning, including related pitfalls and solutions. Lastly, we created a figure to illustrate ideas and end with a general discussion. Our reflective practice questions for UDL rubrics, figure, focus group, and discussion aim to increase positive action for equity, diversity, and inclusion in the classroom and beyond.

## Graphical abstract

The premise of this article, which is a response to a special issue call, “Taking learning online in ecology and evolution," is to promote equity, diversity, and inclusion for large first‐year ecology and evolution lessons. This article provides citations and ideas as well as before, during, and after reflective questions rubrics for universal design for learning, a conceptual figure, and focus group relevant for during the COVID‐19 pandemic. Many ideas provided, however, will be useful after the pandemic is over.

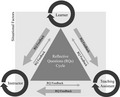

## 1 INTRODUCTION

### 1.1 Equity, diversity, and inclusion are important

Equity, diversity, and inclusion are crucial to 21st‐century higher education and are increasingly discussed, critiqued, and improved with multiple approaches to recognize intersectionalities and enact positive change in work, research, teaching, and learning (Byrd, Brunn‐Bevel, & Ovink 2019). Undergraduate learners come to the classroom as a diverse mosaic with different cultures, talents, disciplinary backgrounds, orientations, life‐stages, and classroom expectations. Instructors can deliberately design for equity, diversity, and inclusion, including for large first‐year classes. Equity, diversity, and inclusion in teaching and learning are frequently discussed on the level of principle and theory, and it is important to translate work for all educators for implementation and practice, including teaching strategies useful to helping across disciplines (Hartwell et al., 2017).

### 1.2 Universal design for learning (UDL) can be implemented in classrooms to foster equity, diversity, and inclusion

Universal design for learning (UDL) is an educational framework that can help design classrooms that are inclusive of all students, especially students often marginalized by regular classroom approaches (Meyer et al., 2014; Sanger, 2020; Schreffler et al., 2019). UDL promotes inclusive pedagogy through multiple means of: (a) engagement (“Why”), (b) representation (“What”), and (c) action and expression (“How”) in order to make lessons more inclusive for all learners (Meyer et al., 2014; Sanger, 2020; Schreffler et al., 2019). In other words, UDL suggests multiple ways for getting students motivated and interested (engagement), acquiring information (representation), and demonstrating what they know (action and expression). This approach embeds equitable thinking and practices into the classroom, making enriching opportunities available to everyone, and in turn, fostering space for all learners to participate.

Especially with large class sizes, fast instruction and lack of scaffolding, many learners in Science, Technology, Engineering and Mathematics (STEM) can face barriers, especially minority students, including those with disabilities; but UDL can support these learners by enabling more than one way to engage, represent, and demonstrate because it helps instructors understand what is the core of the material they are teaching and how material can be adapted for all learners to improve, including those with special needs (Basham & Marino, 2013; Schreffler et al., 2019). Despite the usefulness of the UDL approach, a recent systematic literature review by Schreffler et al. (2019) found that UDL is not commonly used in post‐secondary education in STEM. Basham and Marino (2013) suggest ways in which UDL can be utilized in STEM classrooms, including methods such as increasing variability in teaching by using graphs, simulations and video games to increase different means of engagement, representation as well as action and expression to better teach a diverse group of students with different learning needs. UDL requires planning ahead, to include all types of learners; this planning ahead can lead to relieving stress on STEM instructors and learners because they can be proactive rather than trying to adapt at the last minute (Schreffler et al., 2019). To better plan ahead as well as address on‐the‐spot needs for diverse, equitable, and inclusive classrooms, it helps if all people involved are framing their thinking around being thoughtful and kind; reflective practice is a way to promote such thinking.

### 1.3 Reflective questioning can be implemented in classrooms to foster equity, diversity, and inclusion

Effective practitioners, such as instructors, reflect on what they are doing as professionals throughout their process (Bassot, 2015; Ghaye, 2011; Schon, 1999), and questioning is one way for instructors to reflect on the efficacy of their teaching and learning approaches before, during, and after an action. Critical reflection is a common practice used in the disciplines of general medicine, nursing, social work, and counseling courses, and is helpful for teaching and learning (Bassot, 2015; Ghaye, 2011; Hatzipanagos & Lygo‐Baker, 2006). Reflective practice questioning can be instrumental for the instructor, teaching assistants (TAs), and learners. For the instructor, reflective questioning can encourage them to think about their own biases and proactively strategize how to bring about an inclusive classroom. For the TA, reflective questioning can be a tool to facilitate more engaging discussions with the learners. For the learners, reflective questioning can promote deeper learning and awareness. In this paper we model how reflective practices can facilitate learner agency and the foundation of an inclusive classroom. Reflection is a cornerstone in teaching and learning for adult education (Kolb, 1984). Reflective practice question examples can include any questions, including these appreciative inquiry questions of Ghaye (2011): (a) What is working well? (b) What needs to be changed? (c) What is being learned? (4) Where can we go from here? We explore how reflective questioning, by the instructor, TAs, and learners, can be integrated into a STEM classroom to promote UDL practices, improve course transparency, and ultimately enhance the classroom environment.

## 2 METHODOLOGY

Our methodology is framed around teaching during the pandemic, but can be useful after the pandemic is over. The COVID‐19 pandemic has heightened our awareness of weaknesses in current teaching pedagogies as well as caused much disruption. Our reflective practice questions for UDL in rubrics and the focus group conclusions were developed iteratively through online discussions between the authors. Emphasis was on developing reflective questions to help post‐secondary educators in ecology and evolution science classroom lessons integrate UDL principles. Adding examples to the STEM literature on how to incorporate UDL is of great importance, as mentioned above in the meta‐analysis completed by Schreffler et al. (2019), and we provide one way of doing that by using reflective practice questioning with UDL. UDL in higher education, just like in K‐12, promotes being inclusive of all learners and is perceived by learners without disabilities or with disabilities in post‐secondary as being helpful (Black et al., 2015). The authors assume that educators reading this paper are motivated to improve their student learning, with values that align with improving these spaces through equity, diversity, and inclusion, and pedagogically based approaches.

**Figure 1:**
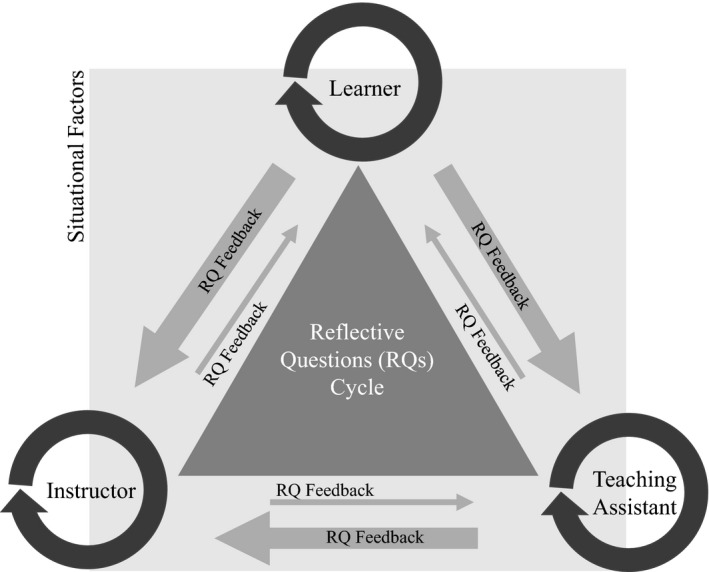
Reflective questions (RQs) cycle for lessons and courses. Reflection, iteration, inclusivity and constructive feedback among instructors, teaching assistants and learners about the course content and learner‐focused needs can foster equity, diversity, and inclusion in classrooms. Different situational factors, such as lived experiences, group dynamics, etc. (Fink, 2007), can arise internally and externally, and checking in intra‐personally and inter‐personally in a reflective and inclusive manner can help with equity, diversity, and inclusion. By promoting both content‐specific skills and equity, diversity and inclusion, key skills to a healthy classroom climate can be fostered: compassion, empathy, and cooperation. The size of the arrows reflects the amount of relative questioning feedback gathered by each stakeholder. Emphasis is on the instructor to gather this feedback, take action, and make the learning environment more conducive to equity, diversity, and inclusion and UDL principles

We focus on the level of ecology and evolution lessons, given a lesson is the level where all involved (learners, TAs and instructors) can daily have an impact, and provide reflective practice question rubrics around UDL to make positive change and support everyone in lesson implementation. This is an iterative process that fosters an environment where everyone involved in the class is impacted by internal and external situational factors, and in which reflective practice questions and UDL can help make the class more equitable, diverse, and inclusive (Figure 1). The rubrics (Tables 1, 2 and 3) act as examples, intended to springboard readers to take, use and modify in other contexts that are best for their classroom needs. A key point to take away from this process (before, during, and after the lesson) is that instructors, TAs, and learners are reflecting internally and expressing ideas with one another externally, with suggestions from learners, instructors, and TAs about lesson components and delivery in a formative way as co‐learners (Figure 1). It is important to also be open to exploring new ways of doing things that might not even be fully tested, as there are many systemic issues that have not been addressed yet in society and education even before the pandemic (Kozleski, 2020).

**Table 1:** Before lesson questions for fostering universal design for learning (UDL) with example reflective practice questions that can be used to promote action (“reflection *for* action,” planning in advance, as it is especially useful during the pandemic to plan ahead; see text for examples)

Instructor	Teaching assistant	Learner
UDL Principle: Multiple Ways of Engagement (Why?)		
Why is this upcoming lesson important? Why will this lesson have these particular learning objectives and goals? Did you check‐in with the TA(s) and learners?	Why is this upcoming lesson important? Why will this lesson have these particular learning objectives and goals? How can you check‐in with the instructor(s) and learners?	Why is this upcoming lesson important to you? Why does this lesson have these particular learning objectives and goals? What relates to your previous knowledge and lived experience? Did you check‐in with the instructor(s) and TA(s)?
UDL Principle: Multiple Ways of Representation (What?)		
What does this upcoming lesson teach? Is information presented in multiple ways? How can you increase representation?	What can you do to support the instructor(s) and learners to be better prepared for engaging in multiple representations?	What representation methods work well for you and what do you need to access to learn well in the lesson? What relates to your previous knowledge and lived experience?
UDL Principle: Multiple Ways of Action and Expression (How?)		
How does this upcoming lesson allow learners to express their knowledge in multiple ways?	How does this upcoming lesson allow learners to express their knowledge in multiple ways?	How does this upcoming lesson allow you to express your knowledge in multiple ways? What relates to your previous knowledge and lived experience? How can you give instructor(s) and TA(s) positive and constructive feedback about assessment of your learning and anything else?

**Table 2:** During lesson questions for fostering universal design for learning (UDL) with example reflective practice questions (“reflection *in* action,” done on the fly and improvised) that can be used to promote action (see text for examples)

Instructor	Teaching assistant	Learner
UDL Principle: Multiple Ways of Engagement (Why?)		
Why is this lesson important? How do you relate the lesson to the learning objectives and goals?	Why is this lesson important? How do you relate the lesson to the learning objectives and goals?	Why is this lesson important? How do you relate the lesson to the learning objectives and goals?
UDL Principle: Multiple Ways of Representation (What?)		
How are learners following your multiple representations? Did you check‐in with the TA(s) and learners?	What are you doing to support the instructor(s) and learners engaging in multiple representations? Did you check‐in with the instructor(s) and learners?	What representation methods are working well for you? Did you check‐in with the instructor(s) and TA(s)? How can you give instructor(s) and TA(s) positive and constructive feedback?
UDL Principle: Multiple Ways of Action and Expression (How?)		
How does this lesson allow learners to express their knowledge in multiple ways?	How does this lesson allow learners to express their knowledge in multiple ways?	Are you able to express your knowledge in multiple ways?

**Table 3:** After lesson questions for fostering universal design for learning (UDL) with example reflective practice questions that can be used to promote action after the lesson (“reflection *on* action”; see text for examples)

Instructor	Teaching assistant	Learner
UDL Principle: Multiple Ways of Engagement (Why?)		
Why was that lesson important? Did learners demonstrate the learning objectives and goals?	Why was that lesson important? Did learners demonstrate the learning objectives and goals?	Why was that lesson important? Did you demonstrate the learning objectives and goals?
UDL Principle: Multiple Ways of Representation (What?)		
Did the learners follow the representations for the learning objectives and goals?	Did the learners follow the representations for the learning objectives and goals?	Did the representations make sense to you in relation to the learning objectives and goals?
UDL Principle: Multiple Ways of Action and Expression (How?)		
How did this lesson allow learners to express their knowledge in multiple ways? Did you check‐in with the learners to help them retain their learning after the lesson? Do the learners need additional support or resources?	How did this lesson allow learners to express their knowledge in multiple ways? Did you check‐in with the learners to help them retain their learning after the lesson? Do the learners need additional support or resources?	Were you able to express your knowledge in multiple ways? Did you check‐in with the instructor(s) and TA(s) to help retain your learning? How can you give instructor(s) and TA(s) positive and constructive feedback? Do you need any additional support? Are there resources outside the classroom (resource center, writing center, etc.) you can use to supplement your learning?

Given STEM culture, which is still not fully inclusive, STEM education includes some learners while excluding other learners, reducing equity, diversity, and inclusion (Simpson & Bouhafa, 2020). We provide solutions and practices to address such inequity in large, first‐year ecology and evolution lessons. We propose that systematically including reflective practice can be used to help instructors, TAs, and students implement and improve equity, diversity, and inclusion measures, and to this end, we provide rubrics with UDL principles and reflective practice to help instructors, TAs, and undergraduate students (henceforth undergraduate students will be referred to as “learners”). We first present examples of reflective practice questions in three rubrics (before, during and after a lesson) aimed at increasing UDL in the classroom that are applicable for large, first‐year courses (applicable to face‐to‐face, online, or blended) and then have a general discussion with ideas from a focus group. The focus group met multiple times in August 2020. While our materials, ideas, and critiques could broadly apply to large first‐year classroom learning, we focus on addressing the question: How do we integrate equity, diversity, and inclusion and UDL into first‐year, undergraduate ecology and evolution introductory lessons given the COVID‐19 pandemic?

## 3 REFLECTIVE PRACTICE AND UDL FOR LESSONS DURING COVID‐19: RUBRICS AND FOCUS GROUP

Regardless of the teaching and learning strategies used (rubric questions, focus group suggestions, etc.), a crucial thing to keep in mind before, during, and after lessons is to be flexible and creative, as there will be no one size fits all; it is key to foster a patient and kind culture, especially given all the COVID‐19 disruptions and changes. Some instructors for spring, summer, and fall 2020 have been forced to re‐write their lesson plans to make them deliverable online; and it is unclear what disruptions might arise due to COVID‐19 even into 2021. Field and laboratory components of ecology and evolution courses are being canceled, deferred, or greatly modified (as they are for research). Furthermore, there are often issues with time and space for instruction; international students, and instructors may be living in different time zones. Being aware and helpful for asynchronous learning is key to many post‐secondary classrooms to accommodate different time zones, which can be complicated.

In spite of teaching and learning being complex and difficult at this time, it is still crucial to incorporate equity, diversity, and inclusion into lessons even with pandemic restrictions. At this time, some institutions will have instruction completely online and others might be blended (online and offline), which will impact how content is taught and learned. While it is difficult (and maybe even contradictory) to unilaterally decide, what an inclusive classroom will look like in first‐year science, biology, or more specifically in ecology and evolution lessons, being thoughtful and reflective and incorporating UDL can help alleviate issues that can arise.

### 3.1 Before lesson rubric: reflection for UDL engagement, representation, action, and expression

Table 1 is a before lesson rubric (reflection *for* UDL action). To motivate and engage (“engagement”, UDL “Why”), learners can be encouraged to be proactive in written or other forms of feedback online. This is their learning journey! Before lesson planning and suggestions can be gathered in multiple ways, including an anonymous online survey or written prompt, to help inform instruction. Planning ahead and being flexible given uncertainty will be common ways to cope during COVID‐19. Instructors can create a culture that fosters learner constructive feedback about what students find helpful or would want to try. Check‐in questions can help with better integration of the types of lived experiences of learners regarding what they want to learn and how they prefer to learn it. Instructors can ask learners via online polls before the lesson preferred presentation methods (give options).

For those instructors who enjoy being outside‐the‐box, they can be creative and think about ways to incorporate STEAM (STEM + the Arts) to foster creative, well‐rounded learners and budding scientists (Segarra et al., 2018). For example, learners if asked in an online poll vote in advance of a lesson might want to do learning in class and why (“engagement”, UDL “Why,” “representation,” UDL “What”) and assessment after class (“action and expression,” UDL “How”) to represent food web networks using offline, online, or blended collage (with pictures of birds, earthworms, etc. from photos, drawings, etc.) and then uploading the artwork for assessment (“action and expression,” UDL “How”) rather than just online presentations and quizzes.

Teaching assistants (TAs) can help the instructor(s) in monitoring and collecting pre‐quiz and pre‐survey data to gather important information on how learners feel about potential representations. For example, a pre‐reading quiz and pre‐class interest survey, both for participation marks, might show that learners are struggling to understand the importance and basic concepts around population dynamics of plants in an alpine meadow (from quiz) and may want to more, given interest (from survey), bridging to health, and environmental sustainability. In which case, the TAs could use their computer and data science skills to summarize that data from these learners to use for a brainstorming session in a TA meeting with the instructor; during that meeting, they could use concepts from the scientific literature to create a small exercise on population dynamics (using the same concepts as for the plants) of city‐dwelling rats to embed the difficult population dynamics material into health and sustainability in cities given overcrowding, the pandemic, and climate change.

TAs can explore with the instructor(s) what is needed to support learners in active learning through a bridging discussion with learners; undergraduates are often more comfortable talking with other students, and TAs are often graduate students. If the before the lesson questions (Table 1) are used throughout the term, learners will be accustomed to providing feedback and that can help TAs act in a bridging role for reflective practice (Tables 1, 2 and 3; Figure 1) and UDL principles (especially UDL “Why,” “What,” How”). It is important, however, that TAs be paid and not overused for their time for any time they allocate to bridging, as they also need to work on their school work, for example, thesis dissertations. Learners can explore what it means to be an active learner—meaning the learner does not just passively learn the material but instead takes an active role in learning (including through reflections from in‐class feedback)—before class to better prepare them for online learning during the COVID‐19 pandemic. Active learning can require more work, especially in the online format, but also can lead to higher retention of content in STEM across all class sizes (Freeman et al., 2014). Learners can also benefit from discussion boards to express ideas for feedback before the lesson.

While ways to modify the before, during, and after lesson rubrics (Tables 1, 2 and 3) are numerous, potential options to figure out ways to change these rubrics for a particular class can be from asking the learners and the TAs what they would like to see, as well as other instructors (as sounding boards) and from visiting professionals trained in teaching and learning, that is, educational consultants at the learning center of that post‐secondary institution and beyond (Raycroft & Flynn, 2020; Seale et al., 2020). There are many online communities and working groups for teaching and learning as well as other resources for online learning techniques (Mahoney & Hall, 2020; Raycroft & Flynn, 2020; Seale et al., 2020), and for general best practices in undergraduate biology teaching and learning both at the course and departmental levels (Branchaw et al., 2020). While deciding which techniques to use might be overwhelming, it is important to reflect on one's teaching philosophy, including beliefs about what makes learning happen, to guide approaches (Laundon et al., 2020; Merkel, 2020). Achieving inclusion during the pandemic will require lots of trial and error, but the new learning that happens can be useful even after the pandemic; many systemic issues are unresolved in society and education even before the pandemic and new thinking can help change how we teach and learn for equity, diversity, and inclusion (Kozleski, 2020). As well, responses by higher education institutions will likely differ, meaning it is important to keep up‐to‐date with current practices and procedures relevant to the situation specific to particular learners, TAs, and instructors; Crawford et al. (2020) mentions the diversity of higher education institutions’ digital pedagogy responses around the world at the start of the pandemic.

### 3.2 During lesson rubric: reflection in UDL engagement, representation, action, and expression

Table 2 is a during lesson rubric (reflection *in* UDL action). A more rewarding during lesson experience can use information from the before lesson check‐ins (Table 1). Learner comments on representations and how they are working (“representation”, UDL “What”) could be directed privately to the TAs fielding these needs with regular consultation with the instructor, only with prior consent of learners. The TAs could take comments in an anonymous aggregate to the instructor, who can make a brief announcement to re‐clarify the group versus individual requirements for the lesson's learning objectives, active learning activities, and subsequent assessment at the start of the lesson and throughout if needed.

Learners also can benefit from discussion boards to express ideas, questions and general thoughts about ecology and evolution concepts, words and graphics during the lesson; in large first‐year classes, there can be a mix of different years of learners, but many of them might be just out of high school (which likely had more structure than post‐secondary) and can benefit from extra scaffolding without COVID‐19, but given the pandemic and so much uncertainty could benefit from more. Earlier experiences suggest that the transition to university of first‐year learners from high school to university was bumpy in Australia early in the pandemic for example (Kyne & Thompson, 2020). However, there were helpful things for STEM learners such as the ability to chat online in live chat, and the amount of questions increased compared to face‐to‐face, suggesting that some of the what would have been quiet learners in‐person were more interactive online (Kyne & Thompson, 2020).

Instructors can have an outside participation component that has to do with general skills, such as observation (e.g., noticing patterns of biodiversity in your neighborhood, using apps for recording local species in parks or other green spaces, and developing hypotheses), and with the during rubric (Table 2), it helps in real time the people involved in the class be reflective if things are working and when they are not working. Citizen science options are often engaging and rewarding for all ages,and there are many new and established platforms for citizen science (Silvertown, 2009; Trouille et al., 2019), and provide options for UDL (“Why,” “What,” “How”) with reflective practice questions to check‐in and support exploration given different learners will interact with citizen science options—even using the same website or app, or simple observations outside their home with no technology but their eyes and a hand lens—in different ways.

With COVID‐19, even with planning, there will likely be much uncertainty during class, including unexpected issues such online platforms not working given an outage or learners, TAs, and/or instructors unexpectedly needing to take time to take care of loved ones. With the reflective questions, learners are given autonomy to provide feedback on their learning experience to the instructor(s) and TA(s) and also can self‐reflect on their learning process, including when unexpected events happen that derail learning in the lesson. Furthermore, mental health needs or other scenarios may need to be addressed unexpectedly, so it is important to support a flexible during the lesson learning environment. COVID‐19 has impacted the mental health of all ages, including university faculty, staff, and students (undergraduate and graduate) and is likely to have more impacts as the pandemic continues (Sahu, 2020).

### 3.3 After lesson: reflection of UDL engagement, representation, action, and expression

After a lesson (reflection *of* UDL action, Table 3) can refer to anytime following the delivery of the content portion of the ecology and evolution lesson and related content activities; for example, this is applicable for the final wrap‐up portion after the content active learning activities and after class. It is helpful to have at least 5 to 10 min at the end to go over the point of a lesson and the next steps. This helps learners solidify and integrate their engagement (UDL “Why”), representations (UDL “What”), and expression and action (UDL “How”). Learners also can benefit from discussion boards to express if they are unclear, and to arrange times to meet using classroom website software if they are working in a group. This is a useful time to see if learners are clear on what they need to remember, learn, etc. for assessment (and if needed accommodation) options they may have for non‐exam written assignments, quizzes, and/or other tests coming up later in the term. This stage can help learners walk away from the lesson with their ideas more consolidated as they have a beginning, middle, and end hopefully with a clear big picture take home message. For those who enjoy being outside‐the‐box, if given time and a clear rubric, they could do expression and action with STEAM (Segarra et al., 2018), such as assignments that involve sketching, drawing, and painting to express what they learned instead of a traditional post‐quiz.

### 3.4 Focus group meetings

We had two focus group meetings in August 2020 with members of our team. We discussed general ideas related to online learning, our rubrics, and figure. We agreed that if reflective practices for UDL as shown in our rubrics and figure are done well in ecology and evolution lessons, they will be highly effective, but potential breakdowns in effectiveness could arise if people are stressed and unprepared to be reflective. Reflection if properly scaffolded, taught, and done regularly becomes easier. Many first‐year learners may not have these skills from high school or be unsure how to learn in post‐secondary. Learners, teaching assistants (TAs), and instructors should do reflection as part of a course in a helpful fashion, for example, as a main overall course learning objective, which can be modeled by the instructor in the beginning of term when discussing explicitly an equitable, diverse, and inclusive course environment. Learners and TAs can be engaged in creating a classroom community agreement in which learners, TAs, and instructors can explicitly discuss, agree, and write down explicitly ways to help people feel valued in the classroom and how to value others; learners are being assessed on course content, but it does help them to have explicit modeling by their TAs and instructor of equity, diversity, and inclusion practices. It also helps to have ways in a kind fashion to call in (privately discuss) and call out (publicly discuss) if equity, diversity, and inclusion are not being respected. Reflection is helpful for learners as it helps them become better at metacognition and other higher order problem solving; knowing what you know and do not know and also how to work well independently and with others are key skills inside and outside the classroom.

Our focus group members thought that UDL with reflective practice online might be in some ways easier and some ways harder than in‐person delivery of large first‐year, ecology, and evolution lessons. For example, there are learners who prefer online learning, and even some learners who enjoy typing responses more than speaking, so they would have an easier time expressing their needs in response to reflective practice for UDL. However, different time zones (asynchronous classes) and lack of technology access for some learners could result in barriers. We think post‐secondary institutions should make it possible for learners to acquire the technological resources they need for free if they are in need. This is something above the course level, but will impact an ecology and evolution lesson online if not remedied. We also thought it important that learners, TAs, and instructors be positive and inclusive with respect to helping learners online who might need accommodations given disabilities or other specific learning needs. For example, it is important that websites and other online materials be accessible to screen readers for those with visual needs or learning disabilities. Some students with anxiety might find online assessment stressful and might need support advocating for what they need to best foster UDL. The key thing is to have an online classroom culture that promotes being reflective and includes everyone in that reflection, as if some people are not engaged it will be less effective, and to make sure people understand the three principles of UDL and have ways to properly integrate them, as things should be authentic! Also, check‐in with people as they should not be forced to reflect if they are not interested. Focus group members thought a way to acquire responses from people with limited effort would be to have quick poll questions that are acted upon right away by others. It is important that people see that their responses lead to action for people to feel that their engagement matters. Just as the principles of UDL promote multiple ways for engagement with course material and how it is presented and assessed, the check‐ins can use different types of methods (anonymous, not anonymous, etc.). Our focus group with respect to online teaching in general had themes about what we thought works well and does not work well. The focus group members concluded the following given online learning: (a) Asynchronous instruction can help learners work with flexibility whenever they can wherever they are as long as they submit their quizzes, assignments, and examinations before the deadline. (b) Multiple representations that were effective have already been implemented (in spring 2020): lecture videos with text of the audio playing at the same time, smaller format audio‐only files, textbooks provided for download, and forums. (c) Forums can be helpful whereby instructors, TAs, and learners answer questions. For large courses, the instructor may not be able to answer all the questions, but for tutorials with a smaller group, for example, forums give a sense of community. (d) Online whiteboard instruction is more difficult because not everyone has a stylus.

## 4 GENERAL DISCUSSION

Given equity, diversity, and inclusion are complex and big topics, we developed reflective practice questions in rubrics (Tables 1, 2 and 3) that highlight engagement, representation, and expression and action of UDL for instructors, teaching assistants, and learners. Rubrics in general are helpful; they can help make things explicit, clarify content assessment, increase grading efficiency, and foster the reflective practice of learners and educators in post‐secondary life sciences classrooms (Allen & Tanner, 2006). Lessons in ecology and evolution with reflective practice questions for UDL that we are advocating for in this paper should also have the same best practices as regular lessons, including both lower‐level and higher‐level thinking as well as active learning activities that link to clear learning objectives (Arievitch, 2020).Being explicit, kind and supportive of instructors, teaching assistants, and learners is even more crucial within the current global COVID‐19 pandemic. Our reflective practice questioning rubrics can work with other educational tools in course design, such as EcoEvo‐MAPS (Summers et al., 2018). Furthermore, starting a course off with clear expectations and fostering an inclusive environment, such as with first‐day information sheets, could help improve equity, diversity, and inclusion practices (Killpack & Melón, 2020) in ecology and evolution classes for the whole semester. It is important for regular check‐ins to promote UDL. Lesson reflective practice (such as questioning) for UDL can help instructors, teaching assistants, and learners have the same understanding of one another's needs before, during, and after lessons overall and with group work. Notwithstanding, difficulty can still arise in spite of regular check‐ins and other best practices. Our team decided to have a focus group to discuss ideas relating to navigating the pandemic with online learning to supplement ideas related to UDL and having classrooms friendly to equity, diversity, and inclusion during the pandemic; we identified pitfalls and strengths of online learning. For our reflective question rubrics and focus group ideas that we provide in this paper, we focus on the level of lesson implementation because this is where individual instructors have the most influence. However, we also acknowledge that curricula development is also necessary to better incorporate UDL and other pedagogies that help inclusion (Meyer et al., 2014; Sanger, 2020; Schreffler et al., 2019) such as culturally responsive teaching (Wlodkowski & Ginsberg, 1995). We encourage those wanting to be more inclusive to do further reading relevant to scales beyond a single lesson and have provided references earlier in the text in previous sections (see above). A framework that might interest such readers is the motivational framework for culturally responsive teaching that respects different individual cultures during learning (Wlodkowski & Ginsberg, 1995), which is complementary to UDL (Kieran & Anderson, 2019). Furthermore, readers can become more familiar with educational pedagogy theory and Scholarship for Teaching and Learning frameworks to gain ideas and confidence (Shawa, 2020; Tierney, 2020). Promoting learner wellbeing and motivation while learning course content during COVID‐19 will require many approaches. With high motivation of learners, there will be better student learning and engagement at the course level. For example, Vroom's expectancy theory (Vroom, 1964) suggests a person will calculate the expected outcomes and apply an effort matched to that outcome. If there are unclear learning outcomes posted online about courses and throughout courses, learners will not be able to accurately calculate the expected outcome of their efforts as they will not know at which level or topics for mastery are and morale would go down. This theory also recognizes that each person will have different priorities in outcomes and direct their efforts accordingly (diversity in priorities). As learners set goals and demonstrate motivation around those goals (Locke, 1968), it is important for course coordinators, administrators, etc. to support learners with the tools they need to make good goals and achieve those goals, etc. Reflective practice and UDL can create an inclusive classroom culture that foster skills that can potentially extend beyond the classroom. Such sharing requires planning to make sure ethics approval is conducted given privacy and human rights of learners, TAs, and instructors. Equitable, diverse, and inclusive classrooms require university support at all levels (including, faculty, staff, and learners) and everyone plays a crucial role. We are adding teaching assistants, often filled by graduate students, to this equation because they are uniquely positioned as mediators. Graduate students can advocate to improve the undergraduate student experience in a classroom. The lived experiences of graduate students can often be much closer to undergraduate student experiences than that of an instructor. These experiences can help bridge the gap between the instructor and student. To leverage this experience, professional development and training should be allocated for this type of work. Everyone at the university would benefit from training in equity, diversity, and inclusion as academic culture often fails to seamlessly embed these into the university landscape (Perez et al., 2020). In creating the reflective practice question rubric and conducting a focus group for lessons in evolution and ecology, we aim to contribute to decreasing the current gaps in diversity in the fields of evolution and ecology (O’Brien et al., 2020).

## CONFLICT OF INTEREST

Authors had no conflicts of interest.

## AUTHOR CONTRIBUTION

**Laura Super:** Conceptualization (lead); Visualization (lead); Writing‐original draft (lead); Writing‐review & editing (lead). **Analise Hofmann:** Conceptualization (lead); Visualization (equal); Writing‐original draft (supporting); Writing‐review & editing (equal). **Connie Leung:** Conceptualization (equal); Visualization (supporting); Writing‐original draft (equal); Writing‐review & editing (equal). **Mabel Ho:** Conceptualization (equal); Visualization (equal); Writing‐original draft (equal); Writing‐review & editing (supporting). **Emma Harrower:** Conceptualization (supporting); Visualization (equal); Writing‐original draft (supporting); Writing‐review & editing (supporting). **Najah Adreak:** Conceptualization (equal); Writing‐original draft (supporting); Writing‐review & editing (supporting). **Zohreh Rezaie Manesh:** Conceptualization (supporting); Visualization (supporting); Writing‐original draft (supporting); Writing‐review & editing (supporting).
